# Supplementary material for: Migratory insights from singing humpback whales recorded around central New Zealand
Source: R Soc Open Sci. 2020 Nov 18;7(11):201084. doi: 10.1098/rsos.201084 (PMC7735341; doi:10.1098/rsos.201084)
Supplement: Supplementary material [file rsos201084supp1.docx]

Migratory insights from singing humpback whales recorded around central New Zealand

- supplementary material

Victoria E. Warren, Rochelle Constantine, Michael Noad, Claire Garrigue, Ellen C. Garland

**S1. Detection of song in New Zealand data**

The PAMGuard (1) whistle and moan detector (2) was applied to all 16 kHz data from the four recording locations. The detector was applied to spectrograms with Fast Fourier Transform (FFT) length 1024, Hann window with 50% overlap, and detected frequency contours between 100 and 600 Hz with an 8 dB threshold, median filter (length 61), average subtraction of 0.02, with minimum connection length of 10 time slices and minimum total size of 20 pixels. The detector outputs were used to direct in-depth examination of the data: data from time periods with detections were thoroughly examined to determine the presence or absence of humpback whale (*Megaptera novaeangliae*) song.

The PAMGuard whistle and moan detector made detections at all four recording locations (**Figure S1.1**). At the Kaikōura and Wairarapa recording locations, a large number of detections were made between November 2016 and the end of the recording period. These were primarily false positive detections arising from the Schlumberger Pegasus Basin 3D seismic survey (10 November 2016 to June 2017) (https://www.doc.govt.nz/our-work/seismic-surveys-code-of-conduct/marine-mammal-impact-assessments/). Outside of the seismic survey, detections were low throughout the recording period at Wairarapa (**Figure S1.1**). The STB detections included false positive detections between 23 October and 2 December 2016 as a result of the PGS Taranaki South 3D seismic survey (https://www.doc.govt.nz/our-work/seismic-surveys-code-of-conduct/marine-mammal-impact-assessments/) (**Figure S1.1**). At this location, further detections during September and the beginning of October were false positive detections of the ‘bioduck’ call from Antarctic minke whales (*Balaenoptera bonaerensis*) (3) (**Figure S1.1**). Confirmed humpback whale song detections occurred during June, July and August (**Figure S1.1**).


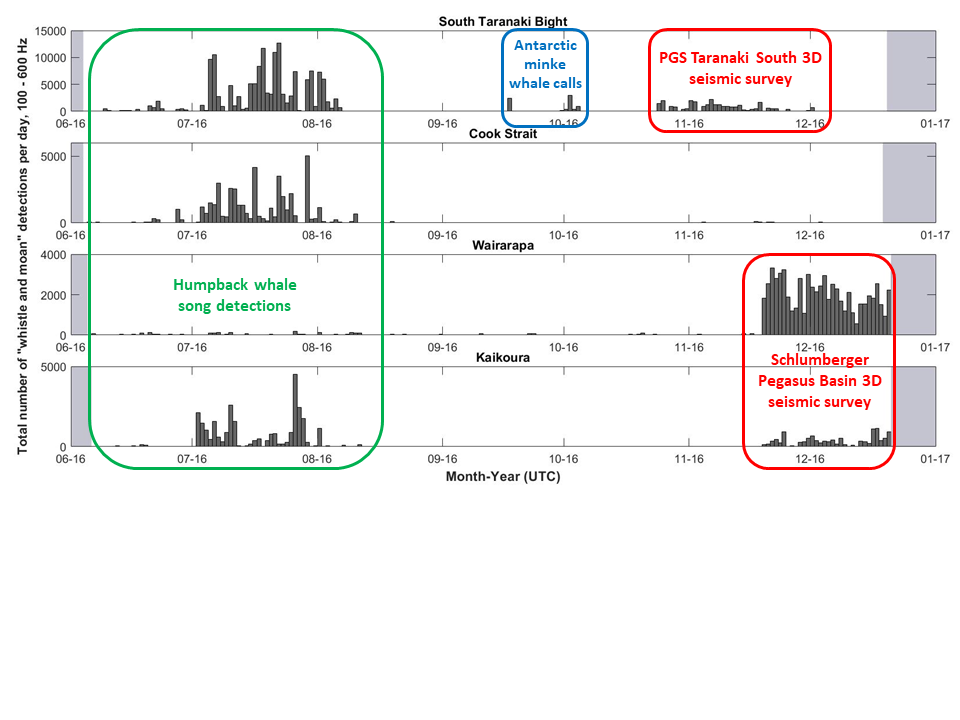


**Figure S1.1**. Raw outputs of the PAMGuard whistle and moan detector for the four central New Zealand recording locations in 2016. Grey blocks indicate times when the acoustic recorders were not deployed. Detections overlaid with red boxes indicate periods of seismic survey activity that resulted in false detections. Detections overlaid with a blue box indicate Antarctic minke whale vocalisations that resulted in false detections. Detections of humpback whale song are overlaid in green, and a verified plot of these detections is provided in Figure 2 of the manuscript. Small numbers of false song detections were made throughout the recording periods with no obvious cause (e.g., September at Wairarapa).

**S2. Consistency of unit classification**

**Table S2.1.** All song units qualitatively described during transcription of the New Zealand, New Caledonia and East Australia songs.

| **Unit short code** | **Full unit name** |
| --- | --- |
| agr(pul) | pulsive ascending groan |
| agr(s) | short ascending groan |
| ahm | ascending high moan |
| ahs | ascending high shriek |
| ahs(l) | long ascending high shriek |
| am | ascending moan |
| am(l) | long ascending moan |
| am(pul) | pulsive ascending moan |
| am(pul)(s) | short pulsive ascending moan |
| am(s) | short ascending moan |
| amm | ascending modulated moan |
| amm(l) | long ascending modulated moan |
| ba | bark |
| ba-hm | bark into high moan |
| ba-w | bark into whoop |
| ba(pul) | pulsive bark |
| chug(l) | long chug-type sound |
| dgr | descending groan |
| dgr(s) | short descending groan |
| dhs | descending high shriek |
| dm | descending moan |
| dm(l) | long descending moan |
| dm(s) | short descending moan |
| dmm | descending modulated moan |
| gr | groan |
| gr-m(pul) | groan into pulsive moan |
| gr-uws | groan into U-shaped whistle |
| gr(l) | long groan |
| gr(s) | short groan |
| gr(s)-dhm | short groan into descending high moan |
| gr(s)-dm | short groan into descending moan |
| gr(s)-dw | short groan into descending whistle |
| gr(s)-mw | short groan into modulated whistle |
| gr(s)-uws | short groan into U-shaped whistle |
| gt(pul) | pulsive grunt |
| gw | growl |
| gw(l) | long growl |
| gw(low) | low growl |
| gw(pul)(l) | long pulsive growl |
| gw(s) | short growl |
| hs | high shriek |
| hs(l) | long high shriek |
| hw | high whistle |
| m | moan |
| m(pul) | pulsive moan |
| m(pul)(l) | long pulsive moan |
| m(pul)(s) | short pulsive moan |
| m(s) | short moan |
| mm | modulated moan |
| mm(l) | long modulated moan |
| mm(pul) | pulsive modulated moan |
| mm(s) | short modulated moan |
| modhs | modulated high shriek |
| modhs(l) | long modulated high shriek |
| mw | modulated whistle |
| mw(l) | long modulated whistle |
| ngr | N-shaped groan |
| ngr(s) | short N-shaped groan |
| ngr(s)-dhm | short N-shaped groan into descending high moan |
| ngr(s)-w | short N-shaped groan into whoop |
| nm | N-shaped moan |
| nm(pul) | pulsive N-shaped moan |
| nsw | N-shaped whistle |
| purr | purr sound |
| purr(l) | long purr sound |
| sealion | sealion barking sound |
| sq | squeak |
| um | U-shaped moan |
| uws | U-shaped whistle |
| w | whoop |
| w-hs | whoop into high shriek |

**Table S2.2. Random Forest variable description and importance measures**

| **Acoustic parameter** | **Description** | **Mean Decrease in Gini Index** |
| --- | --- | --- |
| Duration (s) | Vocalisation length | 208.3 |
| Bandwidth (Hz) | High – Low frequency | 105.5 |
| High frequency (Hz) | Maximum frequency | 102.8 |
| Frequency trend (Hz) | Start / End frequency | 102.7 |
| End frequency (Hz) | End frequency | 97.2 |
| Number of inflections | Number of reversals in slope | 87.9 |
| Low frequency (Hz) | Minimum frequency | 85.1 |
| Start frequency (Hz) | Start frequency | 83.7 |
| Frequency range (Hz) | High / Low frequency | 81.4 |
| Peak frequency (Hz) | Frequency of the spectral peak | 71.2 |
| Pulse repetition rate (per second) | For pulsive sounds | 41.2 |

**Random Forest confusion matrix**

Random Forest confusion matrix included separately as file ‘CSV_S1_RFconfusion.csv’

**S3. Description of phrase types and themes**

**Theme similarity matrix**

Theme similarity matrix included separately as file ‘CSV_S2_PhraseThemeMatrix.csv’

**Table S3.1.** The typical unit sequence (set median) for each phrase type per location and year. Within-set similarity reveals the similarity of unit sequences within a phrase type (between 0, no similarity, and 1, complete similarity). N = sample size. Location key: NZ = New Zealand, NC = New Caledonia, EA = East Australia.

| **Theme** | **Phrase type** | **N** | **Location/year present** | **Set median unit sequence** | **Within-set similarity** |
| --- | --- | --- | --- | --- | --- |
| 1 | 1 | 105 | NZ 2016 | modhs(l), modhs, gw, gw(l) | 0.39 |
|  |  | 21 | NC 2016 | modhs(l), modhs, gw, gw(l) |  |
|  |  | 22 | EA 2015 | hs(l), ahs, gw, gw(l) |  |
| 2 | 2 | 57 | NZ 2016 | am(l), am, gw, gw(l) | 0.83 |
|  |  | 1 | NC 2016 | modhs, am, gw, gw(l) |  |
|  |  | 5 | EA 2015 | am(l), am, gw, gw(l) |  |
| 3 | 3 | 50 | NZ 2016 | dm, w, w, w, w, w, dm, w, w, w, w, w, w, dm | 0.55 |
|  |  | 8 | NC 2016 | dm, w, sq, w, sq, w, sq, w, w, dm, w, w, w, w, w, w, w, dm |  |
|  |  | 42 | NC 2017 | m(pul), sq, w, sq, w, sq, w, m(pul), sq, w, sq, w, sq, w, m(pul) |  |
|  |  | 18 | EA 2015 | dm, w, sq, w, sq, w, sq, w, sq, dm, w, sq, w, sq, w, sq, w, sq, w, sq, dm |  |
|  |  | 71 | EA 2016 | m(pul), sq, w, sq, w, sq, w, m(pul), sq, w, sq, w, sq, w, sq, w, m(pul) |  |
|  |  | 37 | EA 2017 | mm(pul), w, w, w, w, w, w, mm(pul), w, w, w, w, w, w, mm(pul) |  |
|  | 3b | 27 | NC 2017 | m(pul), ba, ba, ba, ba, m(pul), ba, ba, ba, ba | 0.54 |
|  |  | 22 | EA 2016 | m(pul), ba, ba, ba, ba, m(pul), ba, ba, ba, ba, m(pul) |  |
|  |  | 12 | EA 2017 | mm(pul), ba, ba, ba, ba, ba, ba, mm(pul), ba, ba, ba, ba, ba, ba, mm(pul) |  |
| 4 | 4 | 45 | NZ 2016 | dm, ngr(s), ngr(s), ngr(s), ngr(s), dm, ngr(s), ngr(s), ngr(s), ngr(s), ngr(s), dm | 0.66 |
|  |  | 9 | NC 2016 | dm, ngr(s), ngr(s), ngr(s), ngr(s), dm, ngr(s), ngr(s), ngr(s), ngr(s), dm |  |
|  |  | 1 | NC 2017 | nm(pul), ngr(s), ngr(s), ngr(s), m(pul), ngr(s), ngr(s), ngr(s), am(pul) |  |
|  |  | 11 | EA 2015 | dm, ngr(s), ngr(s), ngr(s), dm, ngr(s), ngr(s), ngr(s), ngr(s), dm |  |
| 5 | 5 | 248 | NZ 2016 | dm, gr(s)-uws, ba, ba, gr(s)-uws, ba, ba, gr(s)-uws | 0.28 |
|  |  | 122 | NC 2015B | gr(s), gr(s)-dw, ba(pul), ba(pul), gr(s)-dw, ba(pul), ba(pul), gr(s)-dw |  |
|  |  | 246 | NC 2016 | gr(s)-uws, ba, ba, ba, gr(s)-uws, ba, ba, ba, gr(s)-uws, m |  |
|  |  | 151 | EA 2015 | m, gr(s)-uws, ba, ba, gr(s)-uws, ba, ba, gr(s)-uws |  |
|  | 5b | 35 | NZ 2016 | gr, uws, uws, gr, uws, gw, gw(l) | 0.45 |
|  |  | 3 | NC 2016 | gr(s)-uws, uws, gr(s)-uws, gw, gw(l) |  |
|  | 5c | 169 | NC 2015B | m, ba-hm, ba, ba, ba-hm, ba, ba, ba-hm | 0.41 |
| 6 | 6 | 16 | NZ 2016 | m, am(s), am(s), am(s), am(s), am(s), am(s) | 0.43 |
|  |  | 120 | NC 2015B | m, am(s), am(s), am(s), am(s) |  |
|  |  | 81 | NC 2016 | m, am(s), am(s), am(s), am(s) |  |
|  |  | 55 | EA 2015 | m, am(s), am(s), am(s), am(s) |  |
|  |  | 1 | EA 2017 | am(pul)(s), am(s), am(s), am(s), am(s) |  |
| 7 | 7 | 12 | NZ 2016 | dm, am(pul)(s), am(pul)(s), am(pul)(s), am(pul)(s) | 0.17 |
|  |  | 30 | NC 2015B | am, gw(s), gw(s), gw(s), gw(s) |  |
|  |  | 66 | NC 2016 | m, m(pul), m(pul) |  |
| 8 | 8 | 13 | NZ 2016 | m, sealion, sealion | 0.38 |
|  |  | 29 | NC 2015B | m, chug(l), chug(l) |  |
|  |  | 16 | NC 2016 | m, sealion, sealion |  |
|  |  | 58 | EA 2015 | m, chug(l), chug(l) |  |
| 9 | 9 | 19 | NC 2017 | gr-m(pul), ba(pul), gr-m(pul), mm(l) | 0.39 |
|  |  | 2 | EA 2015 | m(pul), nm, nm, am, nm |  |
|  |  | 7 | EA 2016 | gr-m(pul), ba(pul), gr-m(pul), mm(l) |  |
|  |  | 35 | EA 2017 | m(pul), ba(pul), m(pul), mm(l) |  |
| 10 | 10 | 215 | NC 2017 | gr, nsw, nsw, gr, nsw, gr, gw(low) | 0.59 |
|  |  | 167 | EA 2016 | gr, nsw, nsw, gr, nsw, gr, gw(low) |  |
|  |  | 156 | EA 2017 | gr, mw, nsw, gr, mw, gr, gr(s), gw(low) |  |
| 11 | 11 | 61 | NC 2017 | mm, mm, mm, purr, purr(l) | 0.51 |
|  |  | 29 | EA 2016 | mm, mm, am, purr, purr(l) |  |
|  |  | 19 | EA 2017 | dm, mm, um, purr(l) |  |
| 12 | 12 | 108 | NC 2015A | dgr, m, ba, m, ba, ba, m | 0.33 |
|  | 12b | 67 | NC 2015A | dgr, gw(s), w-hs, w-hs | 0.71 |
| 13 | 13 | 74 | NC 2015A | dgr, m, m | 0.80 |
|  | 13b | 63 | NC 2015A | dgr, mm, w, w, mm | 0.89 |
| 14 | 14 | 70 | NC 2015A | ahm, ahm, gw | 0.57 |
|  | 14b | 23 | NC 2015A | gw(l), w-hs, w-hs | 0.55 |
| 15 | 15 | 14 | NC 2015A | dgr, ngr(s)-dhm, ngr(s)-w, ngr(s)-dhm, ngr(s)-w, ngr(s)-dhm | 0.37 |
| 16 | 16 | 18 | NC 2015B | mw, ba, w, w, gr(s)-mw, w, w, w, gr(s)-mw, w, w, w, w, gr(s)-mw | 0.47 |


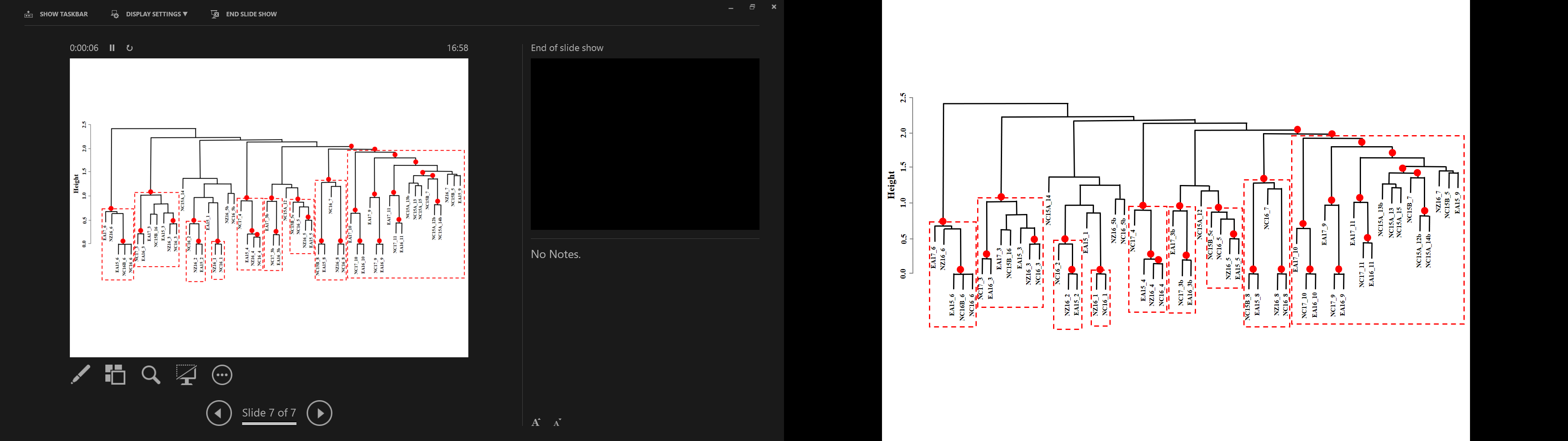


**Figure S3.1.** Bootstrapped (n=1000) dendrogram of average-linkage clustering of the median song strings per phrase type recorded at different locations and years, based on Levenshtein Similarity Index analysis. Red dots indicate AU values >95% where divisions were stable and likely to occur. Red boxes indicate the resulting clusters. The labels are structured as follows: LocationYear_PhraseType.

**S4. Song sequences for each location and year**

Information regarding the themes present at each location/year. The ‘themes/phrase types present’ are the themes that were entered into Dice’s Similarity Index analysis. Set medians per location/year, and per sample were calculated (see ‘Materials and Methods’) to provide an overview of the typical theme sequences and presence.

**New Zealand, 2016**

Themes/phrase types present

1, 2, 3, 4, 5, 5b, 6, 7, 8

Song set median

1, 2, 3, 4, 5, 5b

Song set median, per sample

NZ_STB_X: 1, 2, 3, 4, 5, 5b (n=7)

NZ_STB_Y: 1, 2, 3, 4, 5, 5b (n=5)

NZ_STB_Z: 1, 2, 3, 4, 5 (n=4)

NZ_CS_X: 1, 2, 3, 4, 5, 5b (n=4)

NZ_CS_Y: 1, 2, 3, 4, 5, 5b (n=3)

NZ_CS_Z: 5, 6, 7, 8 (n=7)

**New Caledonia, song A, 2015**

Themes/phrase types present

12, 12b, 13, 13b, 14, 14b, 15

Song set median

12, 13b

Song set median, per sample

NC_15a_X: 13, 12, 14, 15 (n=7)

NC_15a_Y: 12, 13b (n=8)

NC_15a_Z: 12, 13b, 12b, 14b (n=5)

**New Caledonia, song B, 2015**

Themes/phrase types present

5, 5c, 6, 7, 8, 16

Song set median

5c, 6, 8

Song set median, per sample

NC_15b_X: 5, 16 (n=7)

NC_15b_Y: 5c, 6 (n=8)

NC_15b_Z: 5c, 6, 7, 8 (n=9)

**New Caledonia, 2016**

Themes/phrase types present

1, 2, 3, 4, 5, 5b, 6, 7, 8

Song set median

5, 6, 7

Song set median, per sample

NC_16_X: 5, 6, 7 (n=8)

NC_16_Y: 5, 6, 7 (n=9)

NC_16_Z: 5, 6, 7 (n=7)

**New Caledonia, 2017**

Themes/phrase types present

3, 3b, 4, 9, 10, 11

Song set median

3, 3b, 10, 11

Song set median, per sample

NC_17_X: 3, 3b, 10, 11 (n=4)

NC_17_Y: 3, 3b, 9, 10, 11 (n=5)

NC_17_Z: 3, 3b, 10, 11 (n=8)

**East Australia, 2015**

Themes/phrase types present

1, 2, 3, 4, 5, 6, 8, 9

Song set median

5, 6, 8

Song set median, per sample

EA_15_X (northbound): 5, 6, 8 (n=1)

EA_15_Y (northbound): 5, 6, 8 (n=5)

EA_15_Z (southbound): 1, 2, 3, 4, 5 (n=5)

**East Australia, 2016**

Themes/phrase types present

3, 3b, 9, 10, 11

Song set median

3, 3b, 9, 10, 11

Song set median, per sample

EA_16_X: 3, 3b, 9, 10, 11 (n=3)

EA_16_Y: 3, 3b, 9, 10, 11 (n=2)

EA_16_Z: 3, 3b, 9, 10, 11 (n=3)

**East Australia, 2017**

Themes/phrase types present

3, 3b, 6, 9, 10, 11

Song set median

3, 3b, 9, 10, 11

Song set median, per sample

EA_17_X: 3, 3b, 9, 10, 11 (n=5)

EA_17_Y: 3, 3b, 9, 10, 11 (n=4)

EA_17_Z: 3, 3b, 9, 10, 11 (n=5)

**S5. Spectrograms of song types for each location and year**

Spectrograms of the median song strings for each song type recorded per location and year (FFT length 4096, Hann window, 75% overlap, displaying 4 kHz and 140 s, generated in Raven Pro 1.5). Corresponding audio files are provided for each song type (electronic supplementary material, Audio_S1–S10).


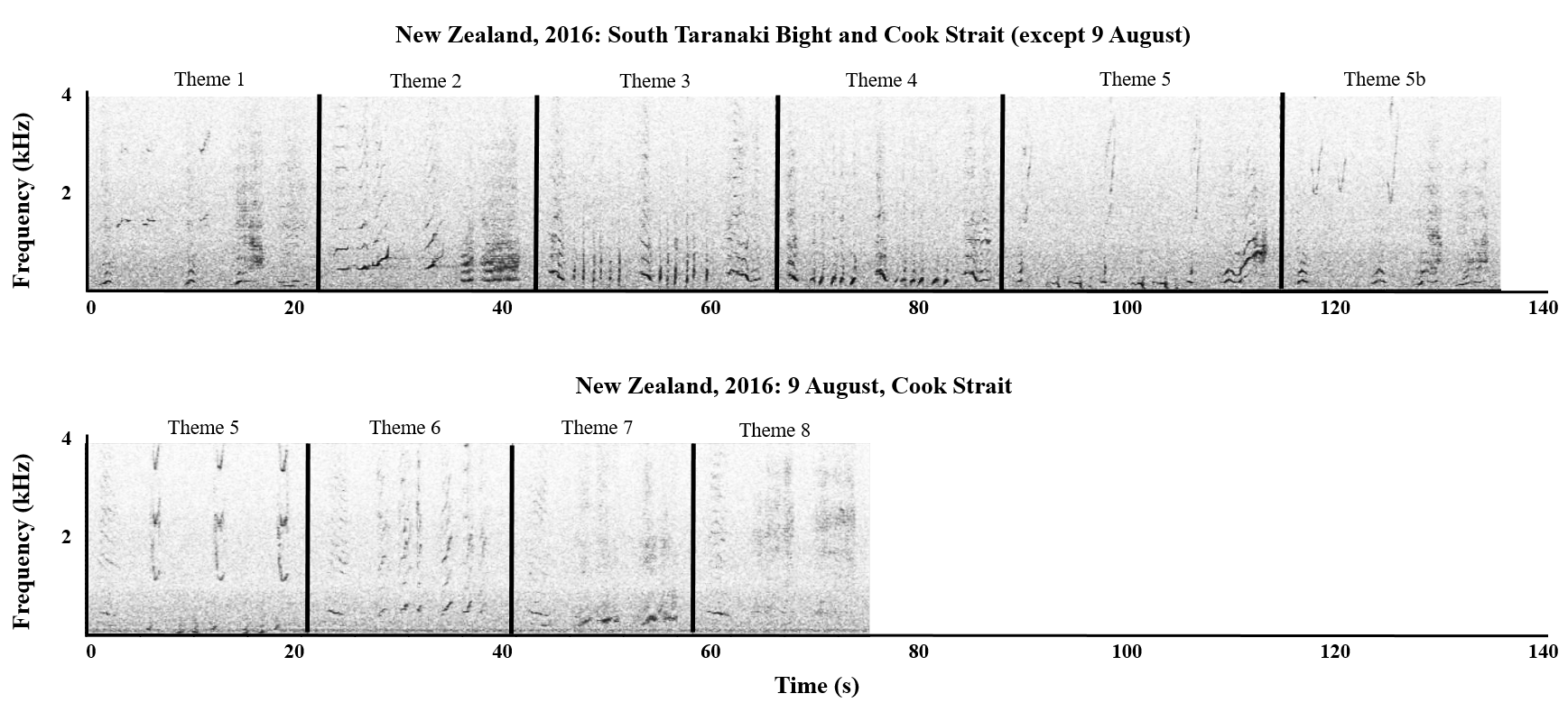


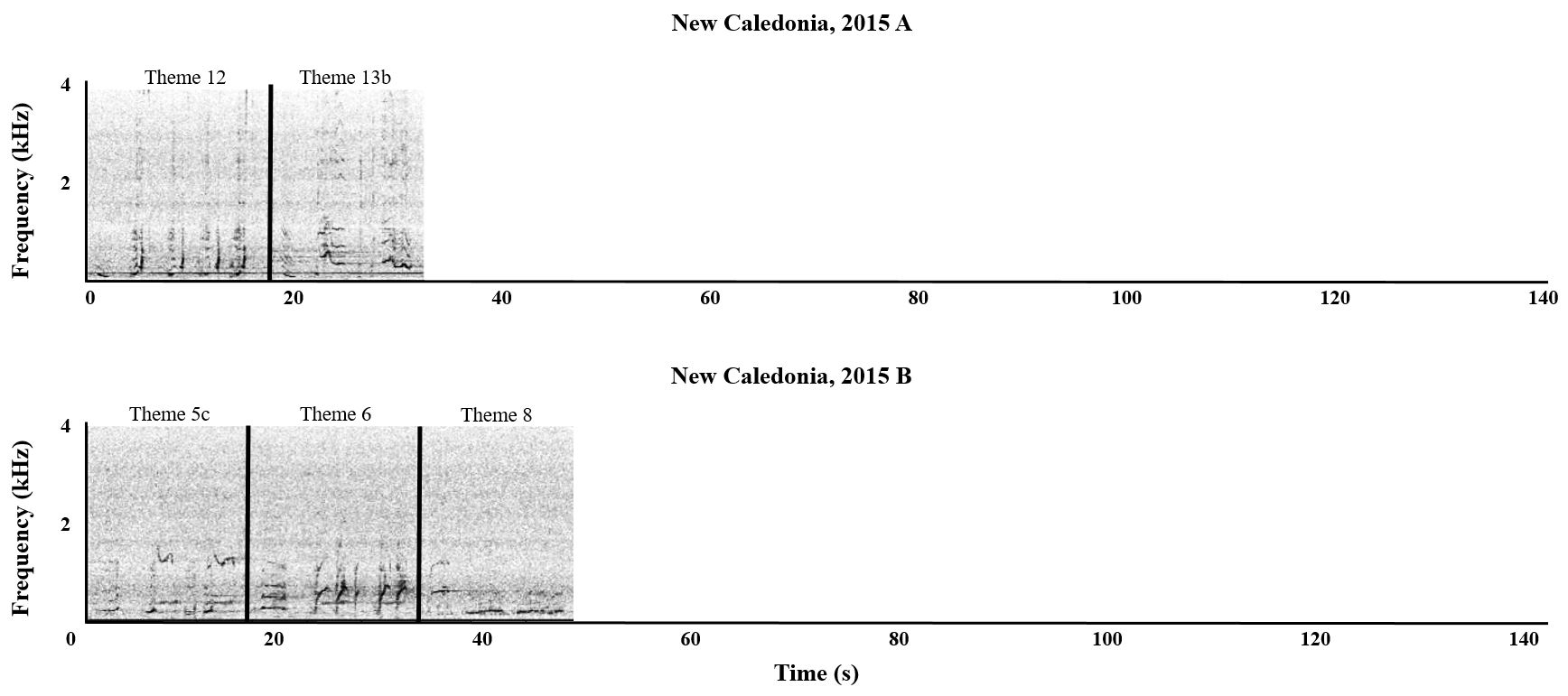


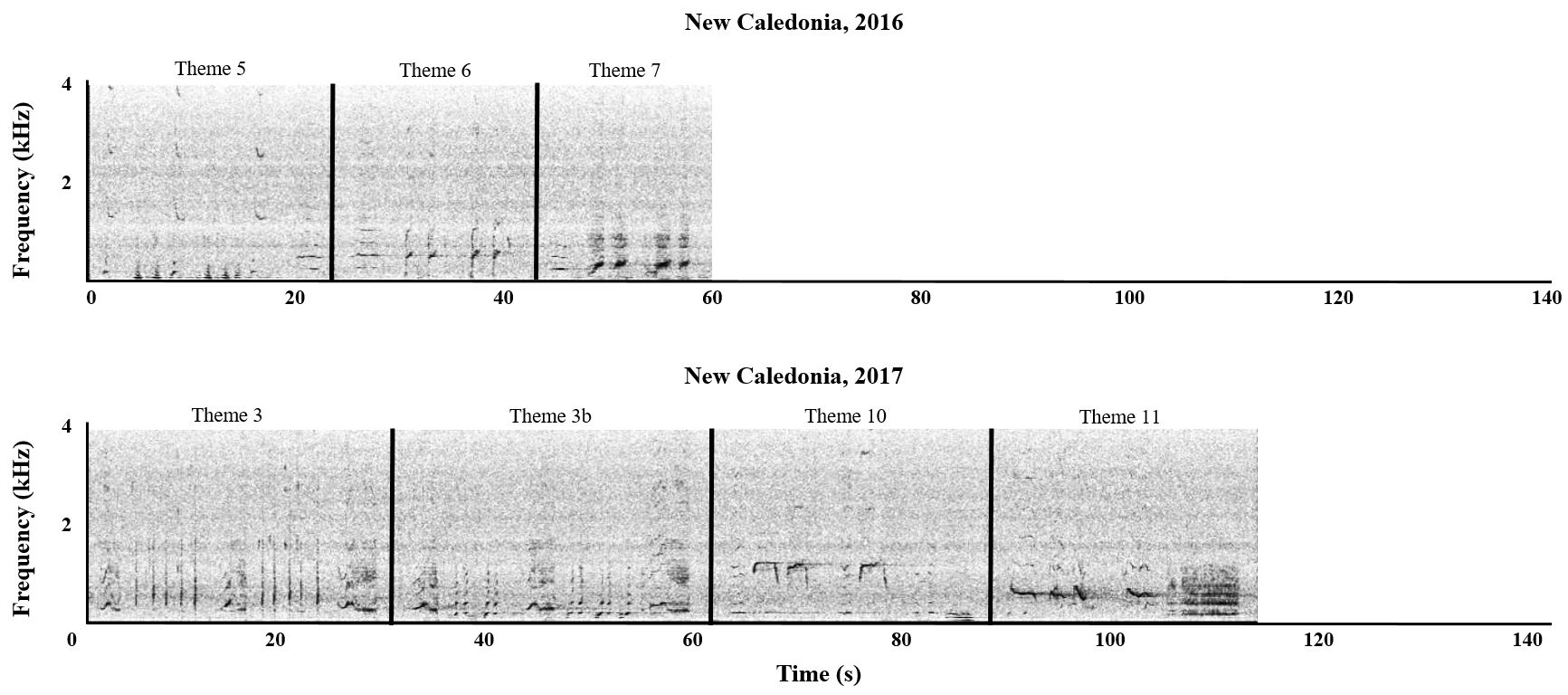


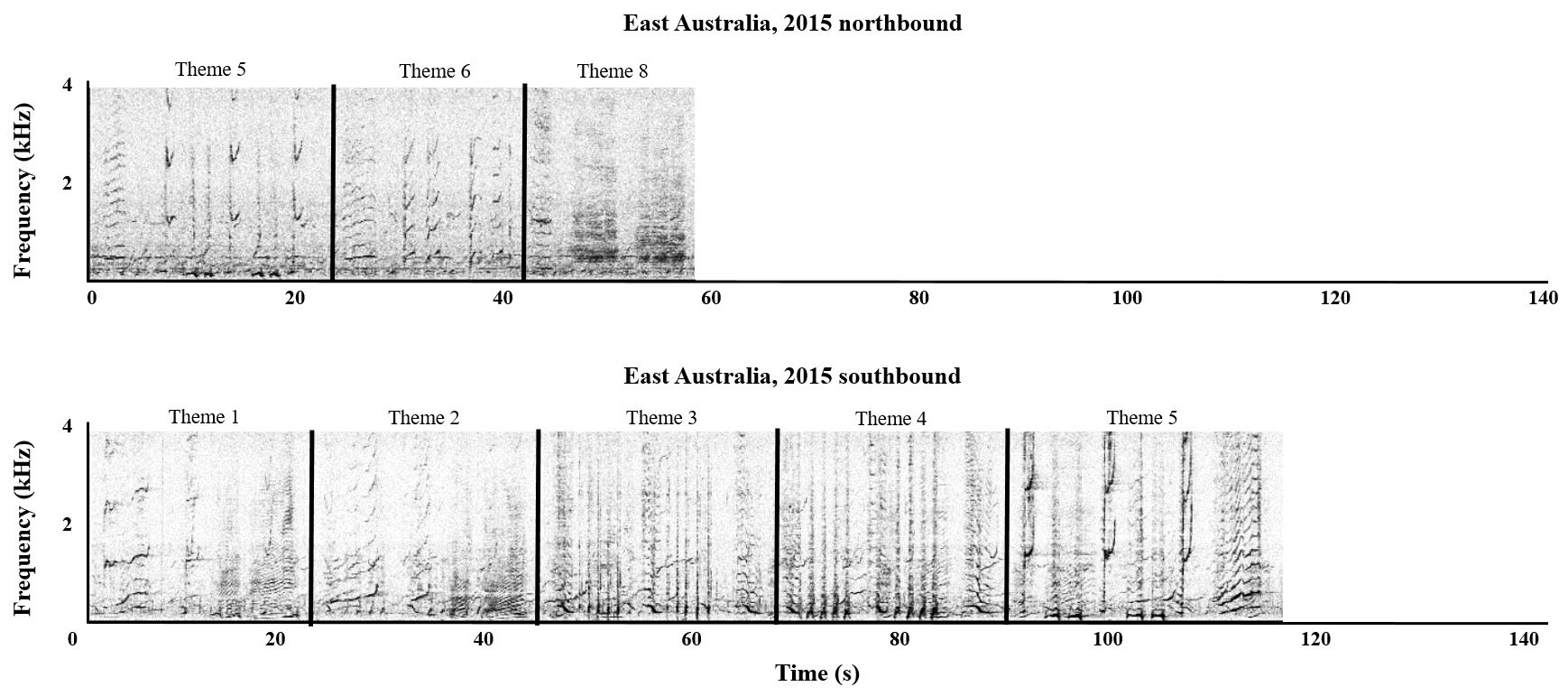


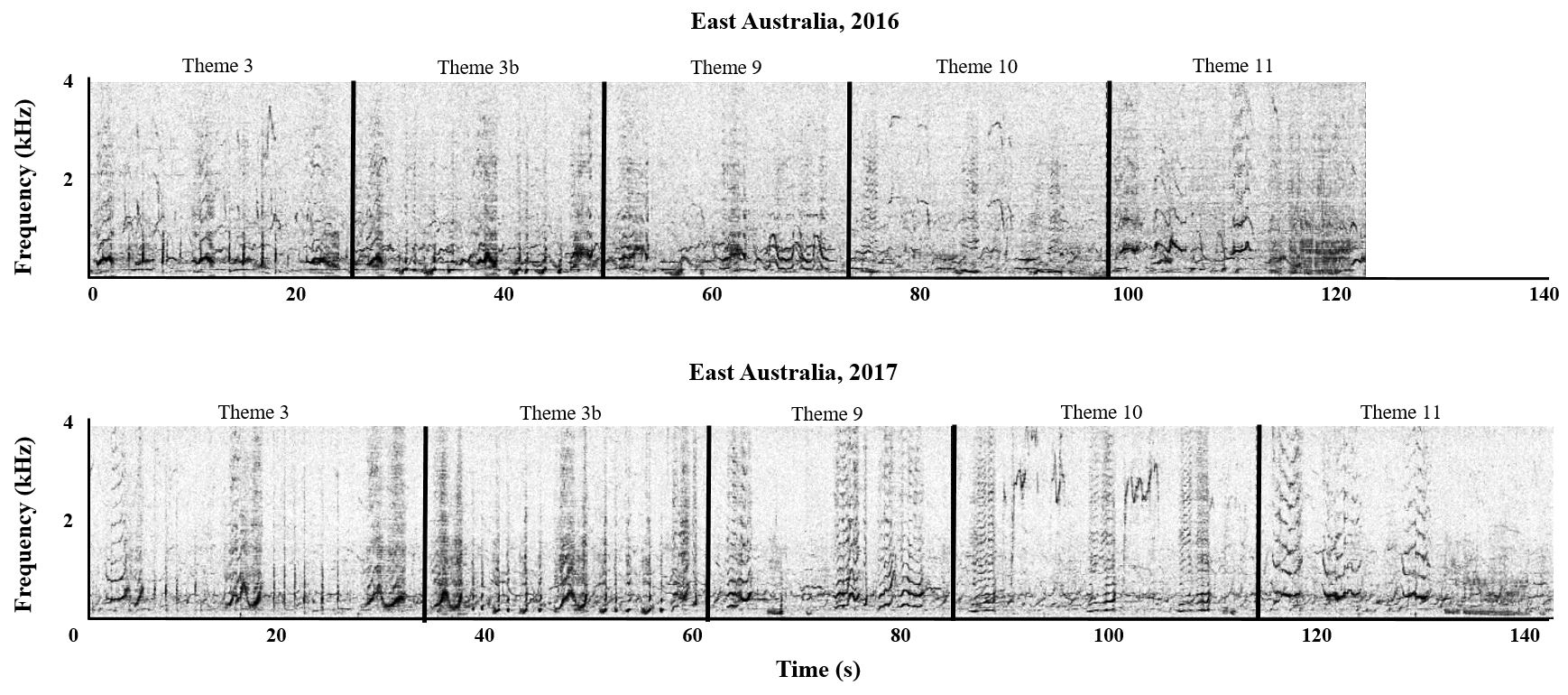


**S6. LSI song matching using the theme similarity matrix**

**Theme similarity matrix**

Theme similarity matrix included separately as file ‘CSV_S3_SongThemeMatrix.csv’


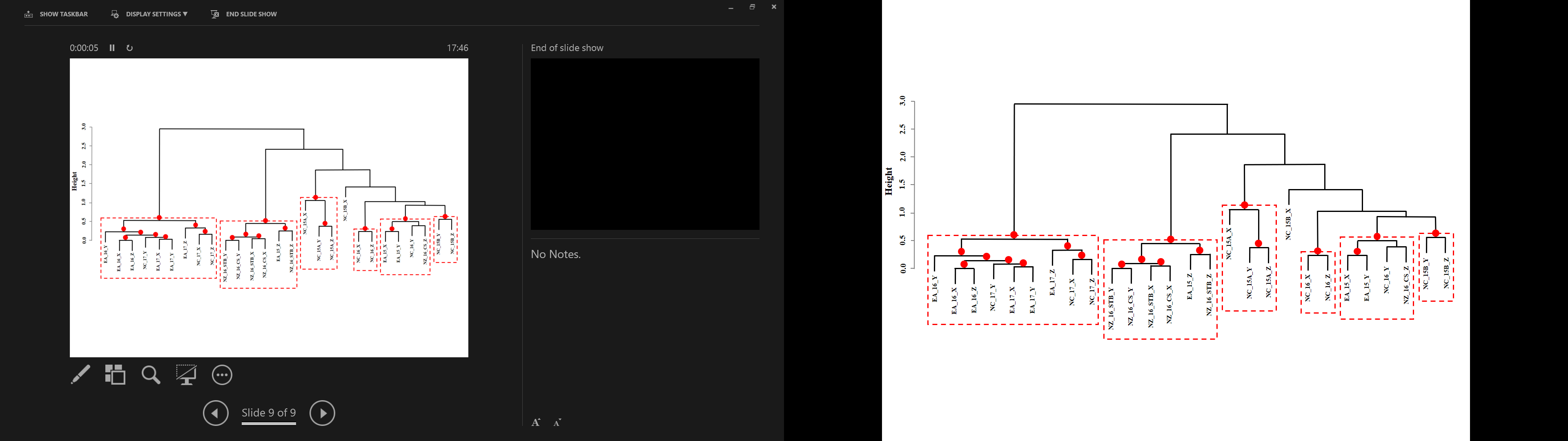


**Figure S6.1.** Bootstrapped (n=1000) dendrogram of average-linkage clustering of the theme similarity matrix of song strings recorded at different locations and years, based on Levenshtein Distance Similarity Index analysis. Red dots indicate AU values >95% where divisions were stable and likely to occur. Red boxes indicate the resulting clusters. The labels are structured as follows: Location_Year_Sub-Location (if applicable)_SongIdentifier. Sub-locations are included for New Zealand 2016: STB = South Taranaki Bight; CS = Cook Strait.

**REFERENCES**

1. Gillespie D, Mellinger DK, Gordon J, McLaren D, Redmond P, McHugh R, et al. PAMGUARD: Semiautomated, open source software for real‐time acoustic detection and localization of cetaceans. J Acoust Soc Am. 2009;125(4):2547.

2. Gillespie D, Caillat M, Gordon J, White P. Automatic detection and classification of odontocete whistles. J Acoust Soc Am. 2013;134(3):2427-37.

3. Risch D, Gales NJ, Gedamke J, Kindermann L, Nowacek DP, Read AJ, et al. Mysterious bio-duck sound attributed to the Antarctic minke whale (*Balaenoptera bonaerensis*). Biol Lett. 2014;10(4).
